# Multicenter retrospective study to evaluate the efficacy and safety of the double‐flap technique as antireflux esophagogastrostomy after proximal gastrectomy (rD‐FLAP Study)

**DOI:** 10.1002/ags3.12216

**Published:** 2018-10-11

**Authors:** Shinji Kuroda, Yasuhiro Choda, Shinya Otsuka, Satoshi Ueyama, Norimitsu Tanaka, Atsushi Muraoka, Shinji Hato, Toshikazu Kimura, Kohji Tanakaya, Satoru Kikuchi, Shunsuke Tanabe, Kazuhiro Noma, Masahiko Nishizaki, Shunsuke Kagawa, Yasuhiro Shirakawa, Yasuaki Kamikawa, Toshiyoshi Fujiwara

**Affiliations:** ^1^ Department of Gastroenterological Surgery Okayama University Graduate School of Medicine, Dentistry and Pharmaceutical Sciences Okayama Japan; ^2^ Department of Surgery Iwakuni Clinical Center Iwakuni Japan; ^3^ Minimally Invasive Therapy Center Okayama University Hospital Okayama Japan; ^4^ Department of Surgery Matsuda Hospital Kurashiki Japan; ^5^ Center for Innovative Clinical Medicine Okayama University Hospital Okayama Japan; ^6^ Department of Surgery Hiroshima City Hiroshima Citizens Hospital Hiroshima Japan; ^7^ Department of Surgery Fukuyama Medical Center Fukuyama Japan; ^8^ Department of Surgery Mihara Red Cross Hospital Mihara Japan; ^9^ Department of Surgery Kagawa Prefectural Center Hospital Takamatsu Japan; ^10^ Department of Surgery Kagawa Rosai Hospital Marugame Japan; ^11^ Department of Surgery Shikoku Cancer Center Matsuyama Japan; ^12^ Department of Surgery Okayama Saiseikai General Hospital Okayama Japan

**Keywords:** antireflux surgery, double‐flap technique, esophagogastrostomy, Kamikawa procedure, proximal gastrectomy

## Abstract

**Aim:**

As a result of the difficulty in effective prevention of gastroesophageal reflux, no standard reconstruction procedure after proximal gastrectomy (PG) has yet been established. The double‐flap technique (DFT), or Kamikawa procedure, is an antireflux reconstruction procedure in esophagogastrostomy. The efficacy of DFT has recently been reported in several studies. However, these were all single‐center studies with a limited number of cases.

**Methods:**

We conducted a multicenter retrospective study in which patients who underwent DFT, irrespective of disease type and reconstruction approach, at each participating institution between 1996 and 2015 were registered. Primary endpoint was incidence of reflux esophagitis at 1‐year after surgery, and secondary endpoint was incidence of anastomosis‐related complications.

**Results:**

Of 546 patients who were eligible for this study, 464 patients who had endoscopic examination at 1‐year follow up were evaluated for reflux esophagitis. Incidence of reflux esophagitis of all grades was 10.6% and that of grade B or higher was 6.0%. Male gender and anastomosis located in the mediastinum/intra‐thorax were independent risk factors for grade B or higher reflux esophagitis (odds ratio [OR]: 4.21, 95% confidence interval [CI]: 1.44‐10.9, *P *=* *0.0109). Total incidence of anastomosis‐related complications was 7.2%, including leakage in 1.5%, strictures in 5.5% and bleeding in 0.6% of cases. Laparoscopic reconstruction was the only independent risk factor for anastomosis‐related complications (OR: 3.93, 95% CI: 1.93‐7.80, *P *=* *0.0003).

**Conclusion:**

Double‐flap technique might be a feasible option after PG for effective prevention of reflux, although anastomotic stricture is a complication that must be well‐prepared for.

## 1 INTRODUCTION

No standard reconstruction procedure after proximal gastrectomy (PG) has yet been established.^1^ PG is mainly indicated for diseases located in the upper‐third of the stomach, such as gastric cancer and submucosal tumor, and recently also for esophagogastric junction (EGJ) cancer. The biggest change caused by PG is loss of the cardia, which plays an extremely important role in antireflux mechanisms, preventing reflux of gastric contents into the esophagus. Although esophagogastrostomy (EG) has the benefits of simplicity and being more physiological, EG with no specific procedure is likely to cause severe reflux esophagitis after surgery, leading to substantial decline in the patient's quality of life (QOL).^2^ Although other reconstruction procedures, such as jejunal interposition (JI),^3^ jejunal pouch interposition (JPI)^4^ and the double‐tract (DT) method,^5^ in which some distance is maintained between the esophagus and gastric remnant, are alternative procedures and are efficient in preventing reflux to some extent, these procedures sometimes cause other problems that are unlikely with EG, such as obstruction of the passage and difficulty in endoscopic surveillance of the gastric remnant after surgery.^6^

The double‐flap technique (DFT), also known as Kamikawa procedure, which was first reported in 1998, is an antireflux procedure during EG after PG. DFT consists of a unique multistep process involving creation of an H‐shaped seromuscular double‐flap, fixing the esophagus and the gastric remnant, and anastomosis and closure of the double‐flap, all of which are basically carried out by hand‐sewn techniques.^7^ In this procedure, the distal esophagus and anastomosis are embedded in the submucosal layer of the gastric remnant and covered by the seromuscular double‐flap, which is designed to function as a one‐way valve to prevent reflux. We previously reported the efficacy of DFT in the reconstruction of antireflux mechanisms, in addition to its ease of performance by laparoscopy through standardization of the procedure and proficiency in laparoscopic suturing and ligation techniques.^7^, ^8^ The efficacy of this procedure has also been reported by other institutions,^9^, ^10^ including reporting of the feasibility of intrathoracic DFT.^11^, ^12^ However, these reports are all based on single institution studies with a limited number of cases, raising concerns about its universality.

In the present study, we aimed to evaluate the universal feasibility of DFT in terms of its efficacy as an antireflux technique and its safety in terms of anastomosis‐related complications by retrospectively collecting DFT cases from multiple institutions. The study population was not limited only to patients with gastric cancer, and other diseases, such as EGJ cancers and gastric submucosal tumor (SMT), were also included. Further, the reconstruction approach was not limited to either laparotomy or laparoscopy, but both approaches were accepted. We believe that this multicenter study with a large number of DFT cases will facilitate the recognition of DFT as a standard reconstruction procedure after PG.

## 2 METHODS

### 2.1 Surgical procedure of DFT

The detailed step‐by‐step procedure and technique of DFT has been described in detail in a previous report.^7^ Briefly, an H‐shaped seromuscular flap (2.5 × 3.5 cm) is first created on the anterior wall of the gastric remnant. The posterior side of the esophagus is fixed by four‐point sutures to the gastric remnant at the upper edge of the flap. Anastomosis of the posterior wall is carried out by a single‐layer continuous suture between all layers of the esophagus and mucosa of the stomach, and anastomosis of the anterior wall is carried out by layer‐to‐layer suturing. The DFT reconstruction is completed by closing the double‐flap in a Y‐shape with interrupted sutures to cover the anastomosis.

### 2.2 Study design

Eighteen institutions participated in the present study. Patients who underwent DFT after PG, irrespective of disease type and reconstruction approach, at each participating institution between January 1st 1996 and December 31st 2015 were retrospectively registered. This study conforms to the provisions of the Declaration of Helsinki, and the protocol was approved by the Okayama University Hospital Institutional Review Board (Approval no. 1705‐023) and the institutional review boards of each participating institution.

### 2.3 Medical records

Characteristics of patients before surgery, including age, gender, height, body weight, body mass index (BMI), prognostic nutritional index (PNI) and disease type, such as gastric cancer, EGJ cancer and gastric SMT, were recorded. In gastric cancer cases, information on histological type, pathological T status (pT), pathological N status (pN) and pathological M status (pM) was also described according to the 3rd English edition of the Japanese Classification of Gastric Carcinoma.^13^ Surgical factors included operation time, blood loss, extent of lymph node dissection, presence of nerve (celiac branch and hepatic branch) preservation, approach for reconstruction, such as laparotomy, thoraco‐laparotomy, mini‐laparotomy and laparoscopy, location of anastomosis (intra‐abdominal or mediastinal/intrathoracic), duration of hospital stay postoperatively and postoperative complications. The approach was considered to be a mini‐laparotomy when reconstruction was carried out through an 8‐cm or shorter skin incision, and it was considered a laparoscopic procedure only when all reconstruction processes were carried out under laparoscopy. At 1‐year follow up, information on reflux esophagitis according to endoscopic examination, as well as body weight, PNI, and regular use of an H2 blocker or proton‐pump inhibitor (PPI) was registered.

### 2.4 Endpoints

Primary endpoint of this study was the incidence of reflux esophagitis approximately 1 year after surgery, which was evaluated using endoscopic examination and scored according to the Los Angeles classification (Grade A to D).^14^ Secondary endpoint was the incidence of anastomosis‐related complications, such as leakage, stricture and bleeding, which were assessed according to the Clavien‐Dindo (CD) classification.^15^ Stricture was considered relevant only when its CD grade was IIIa (requiring balloon dilatation, stenting, or magnetic compression anastomosis) or higher.

### 2.5 Statistical analysis

Statistical analysis was conducted using JMP software ver.10.0.2 (SAS Institute, Cary, NC, USA). Univariate and multivariate logistic regression analyses were carried out to assess the risk factors for reflux esophagitis and anastomosis‐related complications. *P* values < 0.05 were considered statistically significant.

## 3 RESULTS

**Figure 1:**
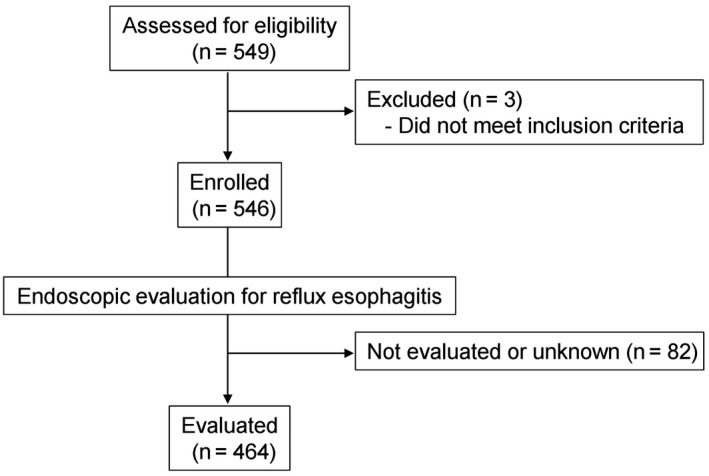
CONSORT diagram for the present study

A total of 546 patients were finally enrolled in the present study from among the 549 cases originally identified after three cases were excluded because of failure to meet inclusion criteria (Figure 1). The primary endpoint was assessed in 464 patients who underwent endoscopic examination approximately 1 year after surgery, whereas the secondary endpoint and other analyses were assessed in the 546 patients.

Gastric cancer was the most common (86.6%) disease registered in this study, followed by EGJ cancer (6.6%), gastric SMT (5.7%) and others (1.1%) (Table 1). Of the 473 gastric cancer cases, differentiated type, pT1, pN0 and pM0 accounted for 74.6%, 78.0%, 89.1% and 98.5% of the cases, respectively (Table S1). In terms of surgical factors, the reconstruction procedure was carried out by laparotomy in 57.0%, thoraco‐laparotomy in 4.6%, mini‐laparotomy in 23.1% and by laparoscopy in 14.8%, and the anastomotic site was located in the abdominal cavity in 92.1% and in the mediastinum/intra‐thorax in 7.9% (Table 2). Celiac and hepatic branches of the vagus nerve were preserved in 46.3% and 70.1% of patients, respectively.

**Table 1:** Characteristics of patients in the present study

Age	
Mean ± SD	68.2 ± 11.1
Gender	
Male	407 (74.5%)
Female	139 (25.5%)
BMI, kg/m^2^	
Mean ± SD	23.0 ± 3.3
PNI	
Mean ± SD	50.6 ± 6.0
Disease	
Gastric cancer	473 (86.6%)
EGJ cancer	36 (6.6%)
Gastric SMT	31 (5.7%)
Others	6 (1.1%)

BMI, body mass index; EGJ, esophagogastric junction; PNI, prognostic nutritional index; SD, standard deviation; SMT, submucosal tumor.

**Table 2:** Surgical factors of patients in the present study

Operation time, min	
Median (IQR)	298 (247.5‐370.5)
Blood loss, mL	
Median (IQR)	240 (100‐392.5)
Lymph node dissection	
D0	43 (7.9%)
D1/1+	487 (89.2%)
Others	16 (2.9%)
Nerve preservation	
Celiac branch	253 (46.3%)
Hepatic branch	383 (70.1%)
Approach for reconstruction	
Laparotomy	311 (57.0%)
Thoraco‐laparotomy	25 (4.6%)
Mini‐laparotomy	126 (23.1%)
Laparoscopy	81 (14.8%)
Others	3 (0.5%)
Location of anastomosis	
Intra‐abdomen	503 (92.1%)
Mediastinum/Intra‐thorax	43 (7.9%)
Postoperative length of hospital stay, days	
Median (IQR)	15 (13‐20)

IQR, interquartile range.

**Figure 2:**
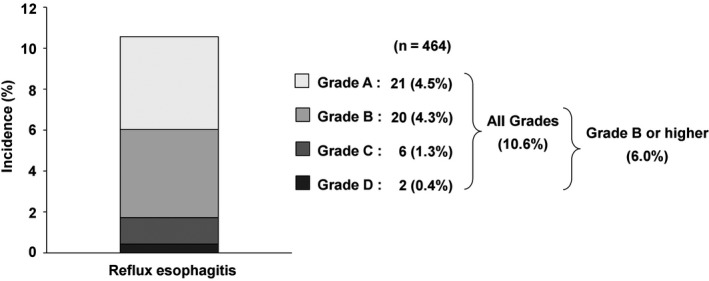
Incidence of reflux esophagitis in the present study

Endoscopic examination carried out at 1.0 year (median) after surgery showed that the incidence of reflux esophagitis, the primary endpoint, was 10.6% for all grades, with an incidence of 4.5% for grade A reflux, 4.3% for grade B, 1.3% for grade C and 0.4% for grade D reflux (Figure 2). The incidence of grade B or higher reflux esophagitis was 6.0%. In gastric cancer cases alone, the incidence of reflux esophagitis was 9.6% for all grades and 4.9% for grade B or higher (Figure S1). Univariate and multivariate analysis showed that anastomosis located in the mediastinum/intra‐thorax was an independent risk factor for grade B or higher reflux esophagitis (odds ratio [OR]: 4.21, 95% confidence interval [CI]: 1.44‐10.9, *P *=* *0.0109), as was male gender (OR: 4.64, 95% CI: 1.35‐29.2, *P *=* *0.0117) (Table 3). When limited to cases in which anastomosis was located in the mediastinum/intra‐thorax, the incidence of reflux esophagitis was as high as 24.3% for all grades and 18.2% for grade B or higher. However, when these cases were historically divided into two groups, the incidence of reflux esophagitis in the late period (2014‐2015) was 11.7% for all grades and 5.9% for grade B or higher, which was substantially reduced compared to 37.5% for all grades and 31.2% for grade B or higher in the early period (1996‐2013). The percentage of patients who regularly took H2 blockers or PPI at 1 year after surgery was 24.7%, and this percentage was significantly higher (47.5%) in patients who suffered from reflux esophagitis at 1 year after surgery (*P *=* *0.0004). Percentage change in body weight at 1 year after surgery in comparison with before surgery was −11.3%, whereas the change in PNI was only −1.8% (Figure S2). There was no correlation between the severity of weight loss and the incidence of reflux esophagitis (*P *=* *0.6776).

**Table 3:** Risk factors for reflux esophagitis

	Univariate	Multivariate		
	*P* value	OR	95% CI	*P* value
Age (≥80 y)	0.4513			
Gender (Male)	0.0120	4.64	1.35‐29.2	0.0117
BMI (≥25 kg/m^2^)	0.2216			
PNI (<45)	0.1192			
Disease (Cancer)	0.8459			
Operation time (≥360 min)	0.6453			
Blood loss (≥500 mL)	0.8482			
Approach to reconstruction (Laparoscopy)	0.3608			
Anastomotic location (Mediastinum/Intra‐thorax)	0.0112	4.21	1.44‐10.9	0.0109
Experience (≤5 cases)	0.8726			

Factors that showed a significant difference in univariate analysis were subjected to multivariate analysis.

BMI, body mass index; CI, confidence interval; OR, odds ratio; PNI, prognostic nutritional index.

**Figure 3:**
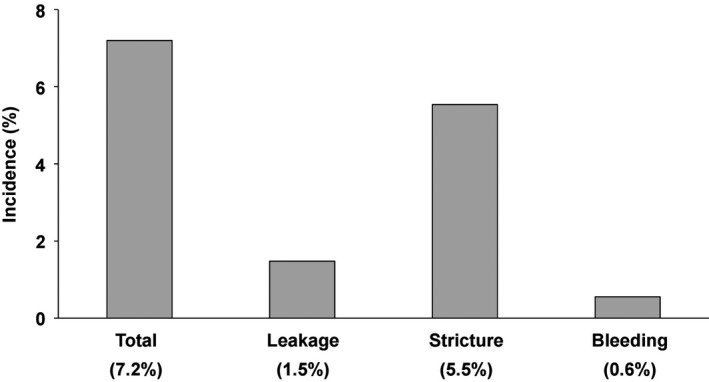
Incidence of anastomosis‐related complications in the present study

The incidence of anastomosis‐related complications, the secondary endpoint, was 7.2% (Figure 3). Strictures were the most frequent complication observed in 5.5% of patients, whereas leakage and bleeding were observed in as few as 1.5% and 0.6% of patients, respectively. All three patients who suffered from anastomotic bleeding had severe blood loss during surgery, whereas no specific finding was observed in eight patients who suffered from anastomotic leakage (Table S2). In gastric cancer cases alone, strictures were observed in 6.0% of patients (Figure S1). Univariate and multivariate analysis showed that laparoscopic reconstruction was the only significant risk factor for anastomosis‐related complications (OR: 3.93, 95% CI: 1.93‐7.80, *P *=* *0.0003) (Table 4). Similarly, when anastomotic stricture was the only complication assessed, laparoscopic reconstruction was the only significant risk factor for development of this complication (OR: 5.53, 95% CI: 2.55‐11.8, *P *<* *0.0001) (Table S3). In an assessment of 81 cases of laparoscopic reconstruction, when the incidence of anastomosis‐related complications was assessed focusing on DFT experience at each institution, the incidence was shown to reduce by approximately 50% after the experience of 11 or more cases (Figure S3).

**Table 4:** Risk factors for anastomosis‐related complications

	Univariate	Multivariate		
	*P* value	OR	95% CI	*P* value
Age (≥80 y)	0.3129			
Gender (Male)	0.2579			
BMI (≥25 kg/m^2^)	0.8444			
PNI (<45)	0.3621			
Disease (Cancer)	0.6498			
Operation time (≥360 min)	0.6377			
Blood loss (≥500 mL)	0.4234			
Approach to reconstruction (Laparoscopy)	0.0003	3.93	1.93‐7.80	0.0003
Anastomotic location (Mediastinum/Intra‐thorax)	0.1410			
Experience (≤5 cases)	0.5233			

Factors that showed a significant difference in univariate analysis were subjected to multivariate analysis.

BMI, body mass index; CI, confidence interval; OR, odds ratio; PNI, prognostic nutritional index.

## 4 DISCUSSION

In the evaluation of reconstruction procedures after PG, the incidence of reflux esophagitis, which is a unique aspect of PG and a major reason why establishment of a standard reconstruction procedure after PG is difficult, is a critical factor determining patients’ long‐term QOL after surgery. Patients who have reflux esophagitis after surgery sometimes suffer from severe symptoms, such as regurgitation, heartburn and cough, for an extended period of time. Simple EG without any additional antireflux procedure has been reported to cause reflux esophagitis in 9.1%‐35.3% of patients, and even JI, JPI and DT, which are supposed to prevent the occurrence of reflux, resulted in reflux esophagitis in 0%‐15.8%, 8.3%‐15.8% and 0%‐25% of cases, respectively, which are not negligible incidences.^2^ Some EG with additional antireflux procedures, such as fundoplication, successfully reduced the incidence of reflux esophagitis to below 10%,^16^, ^17^ whereas other EG failed to prevent the occurrence of reflux even with additional antireflux procedures, resulting in reflux esophagitis in over 30% of cases.^18^, ^19^ Previous DFT reports that included a variety of cases (n = 112) showed that DFT successfully prevented the occurrence of reflux esophagitis (≥grade B) with an incidence of 2.7% (3/112).^7^, ^8^, ^9^, ^10^ In the present study, the incidence of reflux esophagitis was 10.6% for all grades and 6.0% for grade B or higher esophagitis in the analysis of as many as 464 patients, which is considered closer to “real‐world data”. One of the independent risk factors for reflux esophagitis (grade B or higher) was the anastomotic site in the mediastinum/intra‐thorax, which is considered reasonable as a result of the negative pressure of the intrathoracic cavity. Another risk factor was male gender, and this may be related to the fact that gastroesophageal reflux disease, including reflux esophagitis, is more often observed in males than in females.^20^ The biggest advantage of DFT is the effective prevention of reflux symptoms after surgery without the need for additional medications, such as H2 blockers or PPI, which is beneficial for patients and involves reduced treatment costs, although the need for a relatively long time for reconstruction may be a disadvantage. DFT carried out by hand‐sewn techniques only is also associated with cost benefits compared to other reconstruction procedures using stapling devices.

With respect to anastomosis‐related complications, stricture is considered the most frequent complication following DFT that requires careful follow up, whereas leakage is unlikely to occur as a result of the nature of the reconstruction procedure, which involves placement of the anastomosis in the submucosal space by covering it with the seromuscular double‐flap. Previous reports mentioned that anastomotic strictures requiring endoscopic balloon dilatation (CD grade IIIa) occurred in 13.4% (15/112) of patients, whereas anastomotic leakage occurred in only 0.9% (1/112).^7^, ^8^, ^9^, ^10^ In the present study, we showed incidences of anastomotic stricture and anastomotic leakage of 5.5% and 1.5%, respectively, and the only risk factor for anastomosis‐related complications was laparoscopic surgery. DFT was carried out by the laparoscopic (or robotic) approach in over 80% of cases in previous reports, but in only 18.4% of cases in the present study, which may be the reason for the difference in the incidence of anastomotic strictures. Actually, the incidence of anastomotic strictures in the present study was as high as 16.7% (14/84) when the cases with laparoscopic reconstruction were analyzed separately, which is similar to that previously reported. Laparoscopic procedures cannot be avoided both now and in the future as a reconstruction approach after PG for early gastric cancer. As the present study showed, the learning curve was recognized as a factor associated with the incidence of anastomosis‐related complications after laparoscopic DFT, as successful laparoscopic DFT involves certain unique techniques. For this reason, laparoscopic DFT should be carried out by or under the supervision of an experienced surgeon and unprepared introduction of DFT at inexperienced institutions should be avoided.

Although the present study has provided some important information for clinical practice, it has several limitations. First, this was a retrospective study and may have suffered from selection bias. Second, the study spanned a period of 20 years, which is a very long time for a clinical study and may have led to a decline in study quality. Third, evaluation of reflux esophagitis on endoscopic examination was carried out by investigator review, and not by central review, which is advocated worldwide as a means of independent verification of clinical trial endpoints.^21^ Fourth, this study included multiple types of diseases, not only gastric cancer, but also EGJ cancer and SMT, requiring variable extents of lymph node dissection. This difference in extent of lymph node dissection is likely to influence the condition of the anastomotic site, including its blood supply, which has the potential to affect the incidence of both anastomosis‐related complications and reflux esophagitis. From this standpoint, studies targeting a single type of disease requiring the same extent of lymph node dissection may be preferable.

In conclusion, in the present multicenter retrospective study involving a large cohort, the incidence of reflux esophagitis (grade B or higher) with DFT reconstruction after PG was 6.0%. In our opinion, this makes DFT more acceptable as a reconstruction procedure after PG as compared to other procedures. We also showed that the total incidence of anastomosis‐related complications was 7.2%, including anastomotic strictures in 5.5% of cases, which reconfirmed the fact that patients who undergo PG with reconstructive procedures should be carefully followed up for postoperative anastomotic stricture formation, as has also been previously reported. This study is thought to be valuable in providing more universal outcomes than previous reports from single centers involving a limited number of cases. A multicenter prospective study will be required as the next step toward making DFT a standard reconstruction procedure after PG.

## DISCLOSURE

Funding: No financial support was obtained for this study.

Conflicts of Interest: Authors declare no conflicts of interest for this article.

Ethical Statement: This study conforms to the provisions of the Declaration of Helsinki, and the protocol was approved by the Okayama University Hospital Institutional Review Board (Approval no. 1705‐023) and the institutional review board of each participating institution.
