# Supplementary material for: Multicenter retrospective study to evaluate the efficacy and safety of the double‐flap technique as antireflux esophagogastrostomy after proximal gastrectomy (rD‐FLAP Study)
Source: Ann Gastroenterol Surg. 2018 Oct 11;3(1):96–103. doi: 10.1002/ags3.12216 (PMC6345660; doi:10.1002/ags3.12216)
Supplement: Supplementary file 1 [file AGS3-3-96-s001.docx]

**Supporting Information**

Supplementary Table S1. Background and surgical factors for gastric cancer patients

| Age |  |
| --- | --- |
| Mean ± SD | 69.2 ± 10.3 |
| Sex |  |
| Male | 359 (75.9%) |
| Female | 114 (24.1%) |
| BMI, kg/m^2^ |  |
| Mean ± SD | 23.0 ± 3.2 |
| PNI |  |
| Mean ± SD | 50.7 ± 5.9 |
| Histological type |  |
| Differentiated | 350 (74.6%) |
| Undifferentiated | 115 (24.5%) |
| Others | 4 (0.9%) |
| Pathological T status (pT) |  |
| 1 | 366 (78.0%) |
| ≥2 | 103 (22.0%) |
| Pathological N status (pN) |  |
| 0 | 416 (89.1%) |
| ≥1 | 51 (10.9%) |
| Pathological M status (pM) |  |
| 0 | 462 (98.5%) |
| 1 | 7 (1.5%) |
| Operation time, min |  |
| Median (IQR) | 296.5 (249.5 - 365.25) |
| Blood loss, ml |  |
| Median (IQR) | 240 (100 - 400) |
| Lymph node dissection |  |
| D0 | 17 (3.6%) |
| D1/1+ | 444 (93.9%) |
| Others | 12 (2.5%) |
| Nerve preservation |  |
| Celiac branch | 213 (45.0%) |
| Hepatic branch | 339 (71.7%) |
| Approach for reconstruction |  |
| Laparotomy | 282 (59.6%) |
| Thoraco-laparotomy | 2 (0.4%) |
| Mini-laparotomy | 114 (24.1%) |
| Laparoscopy | 72 (15.2%) |
| Others | 3 (0.6%) |
| Location of anastomosis |  |
| Intra-abdomen | 459 (97.0%) |
| Mediastinum/Intra-thorax | 14 (3.0%) |
| Postoperative length of hospital stay, days |  |
| Median (IQR) | 15 (13 - 20) |

SD, standard deviation; BMI, body mass index; PNI, prognostic nutritional index;

IQR, interquartile range.

|  | Age | Sex | BMI, kg/m^2^ | PNI | Disease | Operation time, min | Blood loss, ml | LN dissection | Approach | Location | Experience, case |
| --- | --- | --- | --- | --- | --- | --- | --- | --- | --- | --- | --- |
| Leakage (n=8) | 75 | Male | 27.0 | 58.5 | GC | 241 | 315 | D1 | Laparotomy | Intra-abdomen | 100 |
|  | 74 | Male | 31.7 | 51.6 | GC | 279 | 610 | D1 | Laparotomy | Intra-abdomen | 51 |
|  | 61 | Male | 22.0 | 35.0 | GC | 429 | 115 | D1+ | Mini-laparotomy | Intra-abdomen | 91 |
|  | 62 | Male | 24.7 | 52.0 | GC | 496 | 270 | D1+ | Mini-laparotomy | Intra-abdomen | 102 |
|  | 69 | Female | 22.4 | 53.0 | SMT | 296 | 260 | D0 | Mini-laparotomy | Intra-abdomen | 28 |
|  | 68 | Male | 21.3 | 45.8 | GC | 341 | 10 | D1+ | Laparoscopy | Intra-abdomen | 23 |
|  | 59 | Male | 33.2 | 54.0 | GC | 293 | 260 | D1+ | Laparotomy | Intra-abdomen | 108 |
|  | 61 | Male | 24.0 | 57.6 | GC | 480 | 30 | D1+ | Laparoscopy | Intra-abdomen | 3 |
| Bleeding (n=3) | 77 | Male | 31.5 | 52.2 | GC | 335 | 2500 | D1 | Laparotomy | Intra-abdomen | 5 |
|  | 70 | Male | 21.8 | 46.4 | GC | 464 | 2077 | D1 | Laparotomy | Intra-abdomen | 14 |
|  | 76 | Male | 24.9 | 54.8 | GC | 349 | 1400 | D1+ | Laparotomy | Intra-abdomen | 14 |

Supplementary Table S2. Details of patients who had anastomotic leakage or bleeding

BMI, body mass index; PNI, prognostic nutritional index; LN, lymph node; GC, gastric cancer; SMT, submucosal tumor.

Supplementary Table S3. Risk factors for anastomotic stricture

|  | Univariate | Multivariate | | |
| --- | --- | --- | --- | --- |
|  | *p* value | OR | 95% CI | *p* value |
| Age (≥ 80 years) | 0.6555 |  |  |  |
| Sex (Male) | 0.4833 |  |  |  |
| BMI (≥ 25 kg/m^2^) | 0.5578 |  |  |  |
| PNI (< 45) | 0.3416 |  |  |  |
| Disease (Cancer) | 0.3914 |  |  |  |
| Operation time (≥ 360 min) | 0.6825 |  |  |  |
| Blood loss (≥ 500 ml) | 0.9015 |  |  |  |
| Approach to reconstruction  (Laparoscopy) | <0.0001 | 5.53 | 2.55-11.8 | <0.0001 |
| Anastomotic location  (Mediastinum/Intra-thorax) | 0.2848 |  |  |  |
| Experience (≤ 5 cases) | 0.3890 |  |  |  |

BMI, body mass index; PNI, prognostic nutritional index; OR, odds ratio; CI, confidence interval.

Supplementary Figure S1. Incidence of reflux esophagitis (A) and anastomosis-related complications (B) in gastric cancer cases

Supplementary Figure S2. Body weight and prognostic nutritional index (PNI) before surgery and at 1-year after surgery

Supplementary Figure S3. Incidence of anastomosis-related complications in relation to the learning curve for laparoscopic DFT reconstruction
